# Advances in the Use of DNA Barcodes to Build a Community Phylogeny for Tropical Trees in a Puerto Rican Forest Dynamics Plot

**DOI:** 10.1371/journal.pone.0015409

**Published:** 2010-11-09

**Authors:** W. John Kress, David L. Erickson, Nathan G. Swenson, Jill Thompson, Maria Uriarte, Jess K. Zimmerman

**Affiliations:** 1 Department of Botany, National Museum of Natural History Smithsonian Institution, Washington, D.C., United States of America; 2 Department of Plant Biology, Michigan State University, East Lansing, Michigan, United States of America; 3 Institute for Tropical Ecosystem Studies, University of Puerto Rico, San Juan, Puerto Rico, United States of America; 4 Ecology, Evolution & Environmental Biology, Columbia University, New York, New York, United States of America; Centre National de la Recherche Scientifique, France

## Abstract

**Background:**

Species number, functional traits, and phylogenetic history all contribute to characterizing the biological diversity in plant communities. The phylogenetic component of diversity has been particularly difficult to quantify in species-rich tropical tree assemblages. The compilation of previously published (and often incomplete) data on evolutionary relationships of species into a composite phylogeny of the taxa in a forest, through such programs as *Phylomatic*, has proven useful in building community phylogenies although often of limited resolution. Recently, DNA barcodes have been used to construct a robust community phylogeny for nearly 300 tree species in a forest dynamics plot in Panama using a supermatrix method. In that study sequence data from three barcode loci were used to generate a well-resolved species-level phylogeny.

**Methodology/Principal Findings:**

Here we expand upon this earlier investigation and present results on the use of a phylogenetic constraint tree to generate a community phylogeny for a diverse, tropical forest dynamics plot in Puerto Rico. This enhanced method of phylogenetic reconstruction insures the congruence of the barcode phylogeny with broadly accepted hypotheses on the phylogeny of flowering plants (i.e., APG III) regardless of the number and taxonomic breadth of the taxa sampled. We also compare maximum parsimony versus maximum likelihood estimates of community phylogenetic relationships as well as evaluate the effectiveness of one- versus two- versus three-gene barcodes in resolving community evolutionary history.

**Conclusions/Significance:**

As first demonstrated in the Panamanian forest dynamics plot, the results for the Puerto Rican plot illustrate that highly resolved phylogenies derived from DNA barcode sequence data combined with a constraint tree based on APG III are particularly useful in comparative analysis of phylogenetic diversity and will enhance research on the interface between community ecology and evolution.

## Introduction

Understanding how species are assembled into natural communities in both primary and secondary forests is now a major endeavor of investigation by community ecologists, especially in species-rich tropical biomes [1]–[3]. Species diversity, functional traits (such as wood density, leaf area, and seed size), and evolutionary history (as expressed by phylogenetic relationships) are increasingly under study to determine how species interact with the abiotic environment as well as with other species [4]–[7]. Despite its importance as a metric to describe and compare the structure and diversity of floristic communities, the evolutionary history of a community of species has been particularly difficult to quantify in terms of phylogenetic structure for tropical tree assemblages [7]–[10]. Until recently, community phylogenies were generated from a compilation of previously published (and often incomplete) data on the evolutionary relationships of taxa recorded in a forest plot using programs such as *Phylomatic* [8]. Although this procedure has proven useful in building community phylogenies (see below), they are in many cases incompletely resolved (particular in poorly studied taxa), and therefore, by extension, may have less statistical power than more fully resolved phylogenies to ascertain relationships between functional traits, community ecology, and evolutionary history [11].

Forest dynamics plots, such as the 50-ha plot on Barro Colorado Island (BCI) in Panama, in which all of the woody plant species have been identified, mapped, and periodically censussed [12]–[13] have provided the opportunity to test various conceptual ideas on the rules that govern community assembly, such as the Neutral Theory of Biodiversity [14]. In these plots, the number, diversity, demography, and distribution of species can be quantified and important functional traits and demographic changes can be measured and compared among species and habitats. The evolutionary history, as expressed as a community phylogeny, can also be estimated with varying degrees of accuracy at different levels in the taxonomic hierarchy, e.g., from the ordinal to familial to generic to species-level, and processes of community assembly can be explored using these data. For example, Kembel and Hubbell [15] constructed a community phylogeny of the 312 co-occurring tree species in the forest dynamics plot on BCI based on previously published phylogenies assembled with *Phylomatic*. Their results suggested that tree species in the younger forest and drought-stressed plateaus were phylogenetically clustered, while coexisting species in the swamp and slope habitats were more distantly related than expected. They found on BCI that the average phylogenetic structure was close to random and concluded that in some cases evolutionarily conserved traits were favored in stressful habitats, whereas species composition in the slope and swamp habitats may have been driven by ecological interactions, such as competition.

Insights into community assembly, however, are sensitive to taxonomic resolution. A recent parallel analysis to that of Kembel and Hubbell [15] using DNA barcode sequence data [11] was undertaken to provide a more fully resolved community phylogeny of the species in the BCI plot with the expectation that greater phylogenetic resolution would allow a more detailed understanding of the patterns of community assembly. A DNA barcode is a universally accepted short DNA sequence normally employed for the identification of species [16]–[17]. Sequence data from such species barcodes are now being used for a number of purposes in addition to identification, including the construction of phylogenies [18]. In the study by Kress et al. [11] sequence data from three DNA barcode regions generated for 281 species found in the forest dynamics plot were assembled into a supermatrix to build a nearly completely resolved community phylogeny of the woody trees, shrubs, and palms [11]. Not only was the barcode community phylogeny of BCI plants strongly congruent with the broadly accepted phylogeny of flowering plants [19], but the barcode tree had over 97% of the nodes resolved (Kress et al., 2009), whereas only 48% of the nodes were resolved in the *Phylomatic* tree used by Kembel and Hubbell (2006). This more completely resolved phylogeny led Kress et al. [11] to conclude that of the seven habitat types in the BCI forest dynamics plot, five contained non-random phylogenetic assemblages which in almost every case contradicted the earlier Kembel and Hubbell [15] results based on the *Phylomatic* tree. The finding of Kress et al. [11] demonstrated that less resolved community phylogenies may lead to increased errors in accepting or rejecting null hypotheses of community assembly and structure.

The success of using DNA barcode sequence data in building a community phylogeny of the BCI 50-ha plot has generated considerable interest in applying this methodology to other communities and forest dynamics plots. The complementarity of slowly evolving barcode loci (e.g., *rbcLa*) with faster mutating loci (e.g., *trnH-psbA* and *matK*) together with the application of a supermatrix approach to aligning the sequence data across the widely disparate taxa present in the plot (23 orders, 57 families, and 181 genera) was key to obtaining a well-resolved phylogeny at both the most basal branches as well as the terminal twigs of the tree. The depth and breadth of the species in the BCI study included sufficient taxon sampling to provide significant congruence to the overall angiosperm phylogeny [i.e., 19]. However, for plots with more limited taxon sampling with fewer orders and families, this congruence may not be as robust. Here we expand upon the earlier protocol for generating molecular community phylogenies from DNA barcode sequence data by applying a constraint tree in constructing the phylogeny that is based on the ordinal topology as outlined in the latest Angiosperm Phylogeny Group summary [19]. The Luquillo Forest Dynamics Plot (LFDP) in Puerto Rico, a 16-ha permanent tropical forest plot [20] with lower taxonomic diversity (24 orders, 50 families, 108 genera, and 143 species recorded over multiple censuses of the plot) than BCI, was selected as a trial to test the robustness of the constraint tree approach for building the community phylogeny.

## Results

### DNA Barcode Sequence Recoverability and Quality

PCR and sequencing success were high for both the *rbcLa* region (90.3% of samples; 90.2% of species) and the *trnH-psbA* spacer (83.0% of samples; 92.3% of species); *matK* had the lowest overall rate of recovery (68.8% of samples; 70.4% of species; Table 1). The fractions of bases post trim with Phred scores above 20 were: *rbcLa* = 93%, *matK* = 92%, and *trnH-psbA* = 89%. Overall sequence quality for the *rbcLa* marker was high for all taxa: 94.5% of the successfully amplified taxa were full length and had greater than 50% contig overlap between sequence reads and less than 4% of sequences were partial. For the *trnH-psbA* spacer region 73% of the 131 species with sequence data had contigs with over 50% overlap between sequence reads; the remaining 27% of sequences were interrupted by mononucleotide repeats and had either low overlap between reads or resulted in only partial sequences. Fully 81% of contigs for *trnH-psbA* had full-length sequence when all contigs were included irrespective of sequence overlap. For *matK*, 70% of the 100 recovered species had greater than 50% contig overlap, which was largely due to the length of the amplicon (approximately 850bp using KIM3F_KIM1R primers). Another 15% of *matK* sequences had sequence only in one direction. The fraction of sequences that were full length was not quantified due to difficulty in aligning the primer sequences to the chromatograms.

**Table 1:** Sequence recovery rates for 143 species of trees and shrubs in the Luquillo Forest Dynamics Plot, Puerto Rico.

	DNA Barcode Regions					
Taxonomic Category	*trnH-psbA*	*rbcLa*	*matK*	*rbcLa+trnH-psbA*	*rbcLa+matK*	All
**Samples (n = 288)**	239 (83.0%)	260 (90.3%)	198 (68.8%)	271 (94.1%)	265 (92.0%)	271 (94.1%)
**Species (n = 143)**	131 (92.3%)	129 (90.2%)	100 (70.4%)	136 (95.0%)	128 (90.1%)	136 (95.0%)
**Genus (n = 108)**	106 (98.2%)	106 (98.2%)	76 (70.0%)	107 (99.1%)	106 (98.2%)	107 (99.1%)
**Family (n = 50)**	46 (92.0%)	45 (90.0%)	35 (70.0%)	46 (92.0%)	47 (94.0%)	48 (96.0%)
**Order (n = 24)**	24 (100%)	24 (100%)	22 (91.7%)	24 (100%)	24 (100%)	24 (100%)

For each taxonomic category the absolute number of successes is given along with the percentage relative to the total possible number. In the combinations of markers, sequence success is defined as the recovery of any marker.

### Species Assignment and Identification Using the DNA Barcode Loci

The highest success of species-level assignment using BLAST with individual barcodes was obtained with *matK* (100%), followed by *trnH-psbA* (98%), and then *rbcLa* (93.7%; Table 2). However, when rates of assignment were combined with rates of sequence recovery (i.e., the product of PCR recovery rate with the correct assignment rate), *matK* was less successful, resolving only 70% of the 143 species compared to 83% for *trnH-psbA* and 85% for *rbcLa* (Table 2). Assignment at the level of genus ranged from 70% (*matK*) to 91% (*rbcLa*) and all markers provided 100% correct assignment to family and order (Table 2). The combinations of *matK* and *rbcLa* correctly identified 89% of all species while *trnH-psbA* and *rbcLa* correctly distinguished 93% of all species; the three-locus barcode also identified 93% of the 143species for which sequence data were available.

**Table 2:** BLAST results for frequency of correct identification (CI) for all species and genera.

		Frequency of correct identification (BLAST) per locus		
Taxonomic Rank	Measure	*trnH-psbA*	*rbcLa*	*matK*
**Species (143)**	CI frequency	98%	94%	100%
	Recovery×CI	91%	85%	70%
**Genus (108)**	CI frequency	100%	100%	100%
	Recovery×CI	98%	98%	70%

The number of taxa for each taxonomic level is given in parentheses. For each taxonomic hierarchy the frequency of correct identification for each marker as well as the product of sequence recovery and CI are provided.

### Phylogenetic Reconstruction

#### Maximum parsimony (MP) versus maximum likelihood (ML) analyses

The relatively small amount of sequence data for phylogenetic construction provided by the barcode loci (approximately 520 bp for *rbcLa*, 450 bp for *trnH-psbA*, and 800 bp for *matK*) are expected to result in generally low levels of statistical support (bootstrap or ratchet) for most of the nodes on the trees. For example, 78% of nodes had high levels of support (e.g., >85%) on the trees generated from *rbcLa* alone using maximum parsimony (MP), but only 43.9% for maximum likelihood (ML; Table 3). In the three-locus analyses in which over twice as much nucleotide data were used compared to the single-locus analyses, a little over 88% of the nodes had high support values in the MP tree, while only 50% of the nodes were equally supported in the ML tree. In addition, the relatively sparse sequence data provided by the barcode loci resulted in poorer resolution in the ML trees than in the parsimony trees, no matter how many loci were used.

**Table 3:** Cumulative parsimony ratchet support values for all nodes of each phylogeny generated with sequence data from one to three barcode loci.

Loci Combinations	Support Values					
	<50%	>50%	50–70%	>70%	70–85%	>85%
**Maximum Parsimony**						
***rbcLa***	5.9	94.1	9.8	84.3	6.3	78
***trnH-psbA*** *	3.9	96.1	4.6	91.5	1.5	90
***rbcLa+trnH-psbA***	5.1	94.9	8.4	86.5	3.5	83
***rbcLa+matK***	8.2	91.8	2.8	89	1	88
**3 gene**	8.5	91.5	2.8	88.7	0	88.7
**Maximum Likelihood**						
***rbcLa***	30.2	69.8	16	53.8	9.9	43.9
***rbcLa+trnH-psbA***	22.2	77.8	27	50.8	14.8	36
***rbcLa+matK***	19.2	80.8	26.7	54.1	10.5	43.6
**3 gene**	16.9	83.1	14.8	68.3	18.3	50

Values are given as the percentage of nodes that exhibit a particular range of support level. The total number of species is 143 unless otherwise noted.

*The *trnH-psbA* only tree contained 131 taxa.

This disparity in performance between the two types of analyses may be due in part to the relatively large fraction of missing data for *matK* as ML is often more sensitive to missing data than MP. In addition, ML bootstrap analyses employ a truncated run-time and distort a fraction of the data (with replacement) such that each search of a bootstrap iteration may be less complete. In contrast, the search strategy employed with MP uses the full data matrix for each of the random addition replicates with an exhaustive search among the compiled set of 200 replicates. Thus, the MP search strategy may employ the available sequence data more efficiently than the ML analyses.

#### One- versus two- versus three-locus combinations without a constraint tree

**Figure 1:**
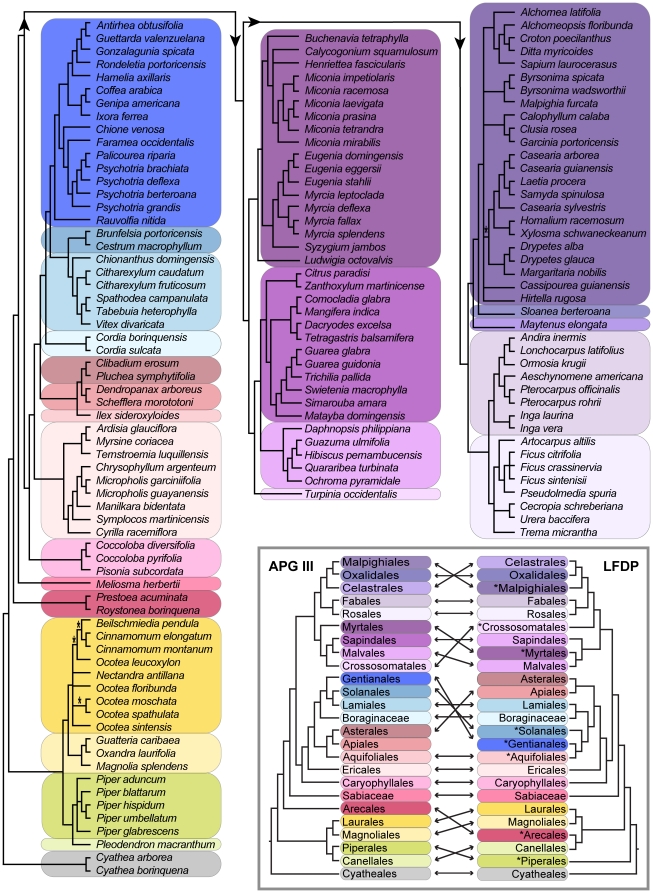
Phylogenetic relationships of the woody plant taxa in the Luquillo Forest Dynamics Plot (LFDP), Puerto Rico. A maximum parsimony consensus tree using an APG III-based ordinal constraint tree combined with a supermatrix of sequence data from the three-locus DNA barcode (*rbcLa*+*trnH-psbA*+*matK*) of the 142 species of woody plants recorded in the LFDP. Taxonomic orders are colored-coded and correspond to orders indicated in inset figure. Nodes with weak support (<70%) are indicated by an asterisk; all other nodes supported at 100%. Comparison of the ordinal topologies of the APG III (2009) consensus phylogeny (left) with the LFDP phylogeny (right) generated without the use of a constraint tree. Arrows connect the position of the same order on each phylogeny. Asterisks indicate those orders that are topologically misplaced in the LFDP phylogeny.

The topology of the MP phylogenetic trees of the LFDP in which no constraint tree was applied were moderately, but significantly incongruent with the accepted ordinal topology (Figure 1; see Inset Figure) as outlined in APG III [19]. The overall topology correctly reflected the basic arrangement of basal angiosperms, lower eudicots, rosids and asterids, but within each of these broader phylogenetic groupings the ordinal relationships varied appreciably from APG III (Figure 1; Inset Figure). In the unconstrained trees constructed with any combination of one to three barcode loci, one-third of the angiosperm orders represented in the forest plot (8 of 24 total orders, including the orphan families Sabiaceae and Boraginaceae) were misplaced with respect to the topology accepted by APG III. This result is in contrast to the BCI barcode phylogeny, which was exceptionally congruent with the APG III topology. The incongruence between BCI and LFDP may be due to increased long-branch attraction if a community, such as the LFDP, is comprised of fewer taxa with increased divergence times among adjacent clades that would complicate phylogenetic reconstruction. We have not tested the effects of these phylogenetic incongruities in the basal branches of the trees on community assembly parameters, such as Net Relatedness Index and Nearest Taxon Index (NRI and N, respectively [4], [11], [21]). However, if these analyses are to be accurate it is necessary for the community phylogeny to provide the best estimate of evolutionary history of the included plant taxa, which according to our results is most efficiently provided by applying a constraint tree.

#### One- versus two- versus three-locus combinations with a constraint tree

Each of the MP trees that were generated using a constraint tree based on APG III [19] from the different combinations of markers (i.e., *rbcLa* alone, *trnH-psbA* alone, *rbcLa*+*trnH-psbA*, *rbcL*a+*matK*, and all three regions combined) differed somewhat in topology, the degree of resolution, and statistical support for nodes (Table 3). Because of the poor sequence recoverability of *matK*, and hence low species sampling, this locus was not used alone to generate a tree. Single-locus trees using *rbcLa* or *trnH-psbA* alone resulted in trees with both polyphyletic families and genera (trees not shown). For example, in the *trnH-psbA* tree at least four families (Euphorbiaceae, Clusiaceae, Urticaceae, and Asteraceae) and four genera (*Ocotea*, *Guarea*, *Psychotria*, and *Casearia*) were polyphyletic. In the tree based on *rbcLa* alone only two families (Euphorbiaceae and Meliaceae) and four genera (*Ocotea*, *Psychotria*, *Eugenia* and *Myrcia*) were polyphyletic. On average more nodes were supported with higher values in the *trnH-psbA* alone tree than in the *rbcLa* alone tree. In both of the two-locus trees (not shown) the resolution was slightly better than the single-locus trees with two families (Euphorbiaceae and Salicaceae) and five genera (*Ocotea*, *Psychotria*, *Casearia*, *Eugenia* and *Myrcia*) polyphyletic. Overall the support values for the two-locus trees were similar to the single-locus trees (Table 3).

The three-locus combination provided the most fully-resolved phylogeny although support at each node was not significantly different than the one and two-locus trees (Figure 1; Table 3). Only a single family (Euphorbiaceae in the Malpighiales [see below]) and four genera (*Ocotea*, *Psychotria*, *Casearia*, and *Myrcia*) were polyphyletic, which may reflect the lack of taxonomic clarity in these taxa. Because of the inclusion of the constraint tree the three-locus tree exactly matched the topology of the APG III ordinal-level phylogeny (Figure 1). Within the specific orders of angiosperms represented in the LFDP, the topology of families as defined on the barcode tree was highly concordant with the APG III classification, except for minor displacements, such as the slightly mis-placed position of the family Rutaceae within the order Sapindales. In some orders, such as the Malpighiales, the lack of resolution among families in the barcode tree parallels the lack of agreement among taxonomists as to family relationships [K. Wurdack, per. comm.; 19]. Finally, 88.7% of the nodes over the entire three-locus MP tree exhibited greater than 85% support values (Table 3).

In a comparison of the three-locus barcode tree with that of the most-up-to-date *Phylomatic* tree for the taxa in the LFDP, the barcode tree has 90.8% of the nodes resolved, whereas only 69.7% of the nodes of the *Phylomatic* tree are resolved.

In summary, the single-locus trees either did not provide enough within-order resolution (*trnH-psbA*), had lower support values (*rbcLa*), or were lacking too many taxa because of problems with sequence recoverability (*matK*). The two-locus trees were better resolved with fewer polyphyletic families, but were not as good as the three-locus tree with respect to resolution and ratchet support values.

## Discussion

### Sequence Recovery and Species Identification for LFDP

In the application of DNA barcodes to the forest dynamics plot on BCI, success in sequence recovery and species identification varied among the three loci. For the BCI taxa, the intergenic spacer *trnH-psbA* and the coding gene *rbcLa* showed the highest percent recovery of species sequences at 94% and 93%, respectively, while *matK* was significantly lower at only 69%. For the LFDP taxa, the frequencies of recovery were almost identical (Table 1) to the results for BCI with *trnH-psbA*, *rbcLa*, and *matK* recovering 92.3%, 90.2%, and 70.4% of the species, respectively. Although the differences in sequence recovery between BCI and LFDP are small, the fewer tissue samples per species available for certain taxa from LFDP may account for the lower probability of recovering a sequence for a particular *species* even though recovery rates per *sample* in most cases may be higher (Table 1).

With regards to correct species assignment using BLAST, two (*trnH-psbA* and *matK*) of the three barcode loci showed similar success in identification of LFDP and BCI taxa for both species and genera at 98–100% and 100%, respectively (Table 2). However, the more conservative, slowly evolving locus *rbcLa* showed significantly higher rates of identification for LFDP (93.7% versus 75% for BCI). The higher rates for *rbcLa* are undoubtedly due to the lower mean number of species per genus and species per family in the Puerto Rican forest than in the Panamanian forest, i.e., 1.31 species per genus and 2.96 species per family in LFDP versus1.64 species per genus and 5.19 species per family on BCI. As first demonstrated for BCI and confirmed here for LFDP even though the frequency of correct identification is highest for *matK*, the lower sequence recovery rates for this locus results in an overall poorer performance in species identification (Table 2).

### Community Phylogeny: Can We Do Any Better With a Constraint Tree?

Our results demonstrate that the application of constraint trees in generating community phylogenies using DNA barcode sequence data is an important step toward ensuring best estimates of evolutionary relationships among the community taxa under study in the forest dynamics plots. *Phylomatic* can generate a relatively well-resolved tree for the LFDP (results not shown), but the barcode tree provides over 20% more fully resolved nodes in the community phylogeny. The amount of data (both sequence data as well as morphological information) required by APG III to determine the ordinal and family relationships of angiosperms is appreciably greater than is normally available for constructing community phylogenies and it is not feasible nor desirable to duplicate these efforts. As demonstrated in the construction of the community phylogeny for the BCI 50-ha plot [11], it is possible to generate a topology that closely parallels that of APG III using DNA barcode sequence data alone. However, as we found with the LFDP, different levels of taxonomic sampling may result in significantly less resolution and less concordance with APG III than desired for investigations of community ecology. The application of a constraint tree at the ordinal level based on the APG III topology has solved this problem. Furthermore, the lower support values and lower levels of resolution in the ML analyses suggest that under these conditions of low amounts of DNA sequence data (and missing data for some loci) it is more feasible to concentrate efforts in MP analyses rather than ML analyses in generating local community phylogenies from DNA barcode sequence data.

Our results for LFDP have shown that from one to three DNA barcode loci can be successfully used in conjunction with the constraint tree to provide significant levels of topological resolution and statistical support for a community phylogeny at the species-level. When all factors are taken into account, such as topological resolution, nodal support values, and the monophyly of families and genera, the three-locus data set (including *rbcLa*+*trnH-psbA*+*matK*) yielded the most fully resolved phylogenetic tree with slightly higher support values than trees based on either one- or two-locus combinations. The combination of *rbcL*+*trnH-psbA* was the second best combination of loci and even *rbcLa* or *trnH-psbA* alone resulted in surprisingly accurate phylogenetic trees (Table 3). In fact the *trnH-psbA* tree surpassed even the three-locus tree if MP support is used as a sole measure of phylogenetic signal. The ability of the more conservative locus *rbcLa* to provide such a highly resolved tree is due in large part to the very low ratio of species to genera in the Luquillo forest (see above).

It would be surprising if more sequence data as provided by the three loci together would have resulted in weaker resolution or lower statistical support for various topologies than a single locus alone. Although the pooling of data can decrease phylogenetic resolution, as where the ILD test detects significant differences, such incongruencies are not common when using data from a single non-recombining plastid genome. An equally important factor for using more loci is that they increase the probability that sequence data will be available for more species in the tree construction because of idiosyncratic problems of sequence recoverability for each of the barcode loci. Nonetheless it is encouraging that even a single barcode locus, such as *trnH-psbA*, combined with a constraint tree will provide a very accurate measure of phylogenetic resolution in a community. Similar investigations in other communities with varying numbers of species will have to strike a balance between available sequence data and the desired levels of resolution and statistical support necessary for a community analysis. Our results suggest that the phylogenetic signal provided by sequence data from the three-locus barcode may be near saturation for phylogenetic analyses of this type and that little will be improved with the addition of more loci in such investigations. More improvement may be gained by increasing the completeness of the three-locus data matrix, i.e., providing missing *matK* sequences, than adding further loci for a subset of the taxa. This result is similar to other barcode studies which have demonstrated that including more than two or three loci does not significantly increase the power of assignment in barcode identifications [22]–[24].

### Conclusion

The use of constraint trees coupled with DNA barcode sequence data in constructing community phylogenies is in essence a combination of a DNA barcode approach as demonstrated by Kress et al. [11] and a *Phylomatic* approach as frequently used in other studies [8], [15]. The constraint tree derived from APG III [19] is identical to the phylogenetic information that would be employed by *Phylomatic* in conjunction with other published phylogenies of the taxa present in the community. In the future when taxonomists have assembled a complete phylogeny for all species of angiosperms, it will be possible to generate a completely resolved community phylogeny with high levels of resolution using an entirely *Phylomatic* approach. Until that time, DNA barcodes will provide not only an accurate forensic tool for studying community ecology, but also a critical phylogenetic tool for exploring community evolution. Highly resolved community phylogenies combined with functional trait and demographic data will enhance our ability to understand the effects of the biotic and abiotic environment on the maintenance of species diversity and ecosystem function in plant communities.

## Materials and Methods

### Description of the Site

The Luquillo Forest Dynamics Plot (LFDP) is a 16-ha permanent forest plot (SW corner *18°20′N*, *65°49′W*; 500 m N-S×320 m E-W) located near El Verde Field Station in the Luquillo Mountains of northeastern Puerto Rico. Vegetation and topography of this area are typical of the tabonuco (*Dacryodes excelsa*) forest zone. The forest is classified as subtropical wet in the Holdridge life zone system [25]. The LFDP encompasses a mix of old growth and secondary forest that has been largely free from human disturbance since the 1940s [20]. The LFDP was established in 1990. Censuses are carried out every five years. All free- standing woody stems equal to or greater than 1 cm diameter dbh in the LFDP are tagged, identified to species, and their dbh measured. In this study 143 species (108 genera, 50 families, and 24 orders) recorded in the plot during one or more demographic censuses were included in the analyses (Table S1).

### DNA Extraction, Amplification, and Sequencing

Field collected tissues preserved through silica gel desiccation were derived entirely from photosynthetic material. Leaf material was disrupted in a Tissuelyzer (Qiagen Cat. # 85210) after which tissues were incubated overnight at 55°C using a CTAB based extraction buffer from AutoGen (Holliston, MA). Following incubation the supernatant was removed and placed in a clean 2ml 96-well plate for submission to an AutoGen 960 DNA extraction robot. DNA extractions were hydrated in 100mM Tris-HCl (pH 8.0) and then transferred to Matrix barcode tubes (MatrixTechnologies Cat. # 3735) and stored at −80°C. Working stocks of DNA were transferred to a microtiter plate, diluted 5× with water and then taken to the PCR laboratory.

### PCR and Sequencing

We used routine PCR, with no more than three attempts per sample to recover a PCR amplicon for all 1,035 samples. The PCR cycling conditions were exactly the same for *rbcLa* and *trnH-psbA* (95°C 3min, (94°C 30sec, 55°C 30sec, 72°C 1min)×35cycles, 72°C 10min) following procedures outlined in Kress and Erickson [26], with *matK* requiring lower annealing temperatures and more cycles (95°C 3min [94°C 30sec, 49°C 30sec, 72°C 1min]×40 cycles, 72°C 10min) following Fazekas et al. [22] and always included DMSO at a final concentration of 5%. Primer pairs for each of the gene regions are listed in Table S2. Successful PCR reactions were purified using a 5× diluted mixture of ExoSap (USB, Cat. # 78201). For sequencing, 2–4ul of the purified PCR was used in a 12ul reaction (0.8ul BigDye terminator sequencing mixture (V3.1; ABI, Cat. 4337457), 2.0ul of a 5× buffer (400u Molar Tris-HCL pH 8.0), 1ul of 1uMolar primer and distilled water to volume). Sequencing of *matK* PCR products included DMSO to a final concentration of 4% in the reaction mixture. Cycling sequencing protocols were the same for all markers, (95°C 15sec [94°C 15sec, 50°C 15sec, 60°C 4min]×30). Following cycle sequencing, products were purified on a column of sephadex and sequence reactions were read on an ABI 3730.

### Sequence Editing, Alignment, and Assembly into a Supermatrix

Recovered trace files for each of the three markers were imported into Sequencher 4.8 (GeneCodes Corp.), trimmed, and assembled into contigs. Each of the three markers was handled differently in alignment. The *rbcLa* marker was aligned in Sequencher 4.8. Alignment was unambiguous due to the absence of indel variation and all *rbcLa* sequences were readily aligned with each other in a global alignment. The global *rbcLa* alignment was then exported from Sequencher as an aligned nexus file.

For alignment of *matK*, sequences were exported individually (i.e. un-aligned) in FASTA file format from Sequencher. We then used transAlign [27] to perform alignment via back-translation. The *matK* sequences (with one per species as available) were aligned simultaneously with each other in this manner and saved as an aligned FASTA file. That aligned *matK* FASTA file was then concatenated onto the *rbcLa* alignment using MacClade [28] to produce a two gene alignment for all taxa.

For *trnH-psbA*, contigs were exported from Sequencher as un-aligned FASTA files. FASTA sequences were partitioned taxonomically for alignment, primarily by family. In cases where only one species per family or order was present in the plot, the *trnH-psbA* sequence of that species was not included in the phylogenetic alignment. Each set of taxonomically structured sequences was then aligned using Muscle [29] with default parameters. A total of thirteen separate taxonomically structured files were generated in this way. Assembly of the different sets of aligned *trnH-psbA* sequences into a supermatrix was achieved by sequentially concatenating them with the *rbcLa+matK* alignment in a supermatrix format as described below [e.g., Figure S1 in 11] again using MacClade. The resulting matrix was very sparse, with more than 94% of the matrix consisting of missing data or gaps. Gaps were not coded and were treated as missing data in phylogenetic reconstruction.

### Phylogenetic Reconstruction

We reconstructed a community phylogeny for LDFP using maximum likelihood (ML) and maximum parsimony (MP) algorithms. Three different marker combinations were examined for performance in phylogenetic reconstruction: *rbcL*+*matK*, *rbcL*+*trnH-psbA*, and *rbcL*+*matK*+*trnH-psbA*. For all combinations of markers, 142 species were included (*Heterotrichum cynosum* in the Asteraceae was excluded because only sequence data for *trnH-psbA* was available), with six sequences of *rbcLa* obtained from GenBank and used in conjunction with our barcode sequences. ML analyses were conducted using RAxML [30] via the CIPRES supercomputer cluster (www.phylo.org). The different locus combinations were partitioned for independent model assessment at each marker. For all combinations of markers a single most likely tree was estimated in addition to running 200–250 bootstrap replicates depending on the marker set. The same gene combinations were used in a MP using PAUP v.4.0 [31] and also run through a local cluster in which we implemented a modification of the parsimony ratchet [32] following Carolan et al. [33]. This resulted in a very large number of equally parsimonious trees, with over 350,000 trees produced for the *rbcLa*+*trnH-psbA* data set and over 250,000 produced for *rbcLa*+*matK*. The three gene matrix produced many fewer trees, approximately 25,000. For both ML and MP trees, a 50% majority tree was constructed and used to quantify overall levels of support for each node within the trees, the rates of well-supported monophylly for taxonomic hierarchies (genus, family, order) and concordance with expected topologies. Analyses were conducted both with and without use of a constraint tree, as described below.

### Application of Constraint Trees

Constraint trees were built such that all taxa were present, but within each order, species topology was not resolved. Thus the topology of the 24 orders on LDFP was specified in accordance with APG III [19], but within each order, species were arrayed as a polytomy. This approach allowed the topology of the species within each order to be resolved with the barcode sequence data, while the ordinal backbone of the tree was defined *apriori*. Constraint trees were implemented in both PAUP and RAxML such that only trees that conformed to the APG III ordinal constraint tree were retained for analysis.

## Supporting Information

Table S1 **List of 143 woody plant species from Luquillo Forest Dynamics Plot included in the study.** GenBank accession numbers are given for the three DNA barcode loci used in the community phylogeny analyses. Sequences taken directly from GenBank that were used in constructing the community phylogeny are marked with an asterisk. (DOC)

Table S2 **Primer pairs for barcode regions *rbcLa*, *matK*, and *trnH-psbA***. (DOC)
