# Supplementary material for: Advances in the Use of DNA Barcodes to Build a Community Phylogeny for Tropical Trees in a Puerto Rican Forest Dynamics Plot
Source: PLoS One. 2010 Nov 9;5(11):e15409. doi: 10.1371/journal.pone.0015409 (PMC2976767; doi:10.1371/journal.pone.0015409)
Supplement: Table S1 — List of 143 woody plant species from Luquillo Forest Dynamics Plot included in the study. GenBank accession numbers are given for the three DNA barcode loci used in the community phylogeny analyses. Sequences taken directly from GenBank that were used in constructing the community phylogeny are marked with an asterisk. (DOC) [file pone.0015409.s001.doc]

| **Taxon** | **Family** |  | ***matK*** | | ***rbcLa*** | ***trnH-psbA*** |
| --- | --- | --- | --- | --- | --- | --- |
| *Aeschynomene americana* L. | Fabaceae |  | n/a | | AF30870* | n/a |
| *Alchornea latifolia* Sw. | Euphorbiaceae |  | n/a | | HM446755 | HM446884 |
| *Alchorneopsis floribunda* Mull. Arg. | Euphorbiaceae |  | HM446655 | | HM446756 | HM446885 |
| *Andira inermis* W. Wright | Fabaceae |  | n/a | | HM446757 | HM446886 |
| *Antirhea obtusifolia* Urb*.* | Rubiaceae |  | HM446656 | | HM446758 | HM446887 |
| *Ardisia glauciflora* [Urb.](http://www.ipni.org/ipni/idAuthorSearch.do;jsessionid=81052DF1B1890BB93B6EDEBE675D5963?id=10993-1&back_page=%2Fipni%2FeditSimplePlantNameSearch.do%3Bjsessionid%3D81052DF1B1890BB93B6EDEBE675D5963%3Ffind_wholeName%3DArdisia%2Bglauciflora%26output_format%3Dnormal) | Primulaceae |  | HM446657 | | HM446759 | HM446888 |
| *Artocarpus altilis* Parkinson | Moraceae |  | HM446658 | | HM446760 | HM446889 |
| *Beilschmiedia pendula* Hemsl*.* | Lauraceae |  | | n/a | GQ981679* | n/a |
| *Brunfelsia portoricensis* Krug & Urb. | Solanaceae |  | HM446659 | | HM446761 | HM446890 |
| *Buchenavia tetraphylla* (Aubl.) R.A. Howard | Combretaceae |  | HM446660 | | HM446762 | HM446891 |
| *Byrsonima spicata* (Cav.) DC. | Malpighiaceae |  | HM446661 | | HM446763 | HM446892 |
| *Byrsonima wadsworthii* Little | Malpighiaceae |  | HM446662 | | HM446764 | HM446893 |
| *Calophyllum calaba* L. | Clusiaceae |  | n/a | | HM446765 | HM446894 |
| *Calycogonium squamulosum* Cogn. | Melastomataceae |  | n/a | | HM446766 | HM446895 |
| *Casearia arborea* (Rich.) Urb. | Salicaceae |  | HM446663 | | GQ981686* | HM446896 |
| *Casearia guianensis* Aubl*.* | Salicaceae |  | n/a | | HM446767 | HM446897 |
| *Casearia sylvestris* Sw. | Salicaceae |  | HM446664 | | HM446768 | HM446898 |
| *Cassipourea guianensis* Aubl. | Rhizophoraceae |  | HM446665 | | HM446769 | HM446899 |
| *Cecropia schreberiana* Miq*.* | Urticaceae |  | HM446666 | | HM446770 | HM446900 |
| *Cestrum macrophyllum* Vent. | Solanaceae |  | HM446667 | | HM446771 | HM446901 |
| *Chionanthus domingensis* Lam. | Oleaceae |  | n/a | | HM446772 | HM446902 |
| *Chione venosa* (Sw.) Urb. | Rubiaceae |  | n/a | | GQ852314* | HM446903 |
| *Chrysophyllum argenteum* Jacq. | Sapotaceae |  | HM446668 | | HM446773 | HM446904 |
| *Cinnamomum elongatum* (Nees) Kosterm. | Lauraceae |  | HM446669 | | HM446774 | HM446905 |
| *Cinnamomum montanum* J. Presl | Lauraceae |  | HM446670 | | HM446775 | HM446906 |
| *Citharexylum caudatum* L. | Verbenaceae |  | HM446671 | | HM446776 | HM446907 |
| *Citharexylum fruticosum* L*.* | Verbenaceae |  | HM446672 | | HM446777 | HM446908 |
| *Citrus paradisi* Macfad. | Rutaceae |  | n/a | | HM446778 | HM446909 |
| *Clibadium erosum* (Sw.) DC*.* | Asteraceae |  | HM446673 | | HM446779 | HM446910 |
| *Clusia rosea* Jacq. | Clusiaceae |  | n/a | | HM446780 | HM446911 |
| *Coccoloba diversifolia* Jacq*.* | Polygonaceae |  | HM446674 | | HM446781 | HM446912 |
| *Coccoloba pyrifolia* Desf*.* | Polygonaceae |  | HM446675 | | n/a | HM446913 |
| *Coffea arabica* L. | Rubiaceae |  | HM446676 | | HM446782 | HM446914 |
| *Comocladia glabra* Spreng. | Anacardiaceae |  | HM446677 | | HM446783 | HM446915 |
| *Cordia borinquensis* Urb. | Boraginaceae |  | HM446678 | | HM446784 | HM446916 |
| *Cordia sulcata* DC. | Boraginaceae |  | HM446679 | | n/a | HM446917 |
| *Croton poecilanthus* Urb. | Euphorbiaceae |  | HM446680 | | HM446785 | n/a |
| *Cyathea arborea* (L.) Sm. | Cyatheaceae |  | n/a | | HM446786 | HM446918 |
| *Cyathea borinquena* Domin. | Cyatheaceae |  | n/a | | HM446787 | HM446919 |
| *Cyrilla racemiflora* L. | Cyrillaceae |  | HM446681 | | HM446788 | HM446920 |
| *Dacryodes excelsa* Vahl. | Burseraceae |  | n/a | | HM446789 | HM446921 |
| *Daphnopsis philippiana* Krug. & Urb. | Thymelaeaceae |  | HM446682 | | HM446790 | HM446922 |
| *Dendropanax arboreus* (L.) Decne. & Planch*.* | Araliaceae |  | HM446683 | | HM446791 | HM446923 |
| *Ditta myricoides* Griseb. | Euphorbiaceae |  | HM446684 | | HM446792 | HM446924 |
| *Drypetes alba* Poit. | Euphorbiaceae |  | n/a | | HM446793 | HM446925 |
| *Drypetes glauca* Vahl*.* | Euphorbiaceae |  | n/a | | HM446794 | HM446926 |
| *Eugenia domingensis* O. Berg*.* | Myrtaceae |  | HM446685 | | HM446795 | HM446927 |
| *Eugenia eggersii Kiaersk* | Myrtaceae |  | HM446686 | | HM446796 | HM446928 |
| *Eugenia stahlii* (Kiaersk.) Krug & Urb*.* | Myrtaceae |  | HM446687 | | HM446797 | HM446929 |
| *Faramea occidentalis (L.) A. Rich.* | Rubiaceae |  | HM446688 | | HM446798 | HM446930 |
| *Ficus citrifolia* Mill. | Moraceae |  | HM446689 | | HM446799 | HM446931 |
| *Ficus crassinervia* Desf. | Moraceae |  | HM446690 | | HM446800 | HM446932 |
| *Ficus sintenisii* Warb. | Moraceae |  | HM446691 | | HM446801 | HM446933 |
| *Garcinia portoricensis* (Urb*.*) Alain | Clusiaceae |  | n/a | | HM446865 | HM446995 |
| *Genipa americana* L*.* | Rubiaceae |  | HM446692 | | HM446802 | HM446934 |
| *Gonzalagunia spicata* (Lam.) M. Gomez | Rubiaceae |  | HM446693 | | HM446803 | HM446935 |
| *Guarea glabra* Vahl | Meliaceae |  | HM446694 | | HM446804 | HM446936 |
| *Guarea guidonia* (L.) Sleumer | Meliaceae |  | HM446695 | | HM446805 | HM446937 |
| *Guatteria caribaea* Urb. | Annonaceae |  | HM446696 | | HM446806 | HM446938 |
| *Guazuma ulmifolia* Lam. | Malvaceae |  | n/a | | HM446807 | HM446939 |
| *Guettarda valenzuelana* A. Rich. | Rubiaceae |  | HM446697 | | HM446808 | HM446940 |
| *Hamelia axillaris* Sw. | Rubiaceae |  | n/a | | HM446809 | n/a |
| *Henriettea fascicularis* (Sw.) M. Gomez | Melastomataceae |  | HM446698 | | HM446810 | HM446941 |
| *Heterotrichum cymosum* (J.C. Wendl. ex Spreng.) Urb. | Asteraceae |  | n/a | | n/a | HM446942 |
| *Hibiscus pernambucensis* Arrunda | Malvaceae |  | HM446699 | | HM446811 | HM446943 |
| *Hirtella rugosa* Thuill ex Pers*.* | Chrysobalanaceae |  | n/a | | HM446812 | HM446944 |
| *Homalium racemosum* Jacq. | Salicaceae |  | HM446700 | | HM446813 | HM446945 |
| *Ilex sideroxyloides* Griseb. | Aquifoliaceae |  | n/a | | L01928.2* | n/a |
| *Inga laurina* (Sw.) Willd. | Fabaceae |  | HM446701 | | HM446814 | HM446946 |
| *Inga vera* Willd. | Fabaceae |  | HM446702 | | HM446815 | HM446947 |
| *Ixora ferrea* (Jacq.) Benth. | Rubiaceae |  | HM446703 | | HM446816 | HM446948 |
| *Laetia procera* (Poepp.) Eichler | Salicaceae |  | HM446704 | | HM446817 | HM446949 |
| *Lonchocarpus latifolius* DC. | Fabaceae |  | HM446705 | | HM446818 | HM446950 |
| *Ludwigia octovalvis* (Jacq.) P.H. Raven | Onagraceae |  | n/a | | L1022* | n/a |
| *Magnolia splendens* Urb. | Magnoliaceae |  | n/a | | HM446819 | HM446951 |
| *Malpighia furcata* Ker Gawl. | Malpighiaceae |  | HM446707 | | HM446820 | HM446952 |
| *Mangifera indica* L. | Anacardiaceae |  | n/a | | HM446821 | HM446953 |
| *Manilkara bidentata* (A. DC.) A. Chev*.* | Sapotaceae |  | HM446708 | | HM446822 | HM446954 |
| *Margaritaria nobilis* L.f*.* | Euphorbiaceae |  | HM446709 | | HM446823 | HM446955 |
| *Matayba domingensis* (DC.) Radlk*.* | Sapindaceae |  | HM446710 | | HM446824 | HM446956 |
| *Maytenus elongata* (Urb.) Britton | Celastraceae |  | HM446711 | | HM446825 | HM446957 |
| *Meliosma herbertii* Rolfe | Sabiaceae |  | HM446712 | | HM446826 | HM446958 |
| *Miconia impetiolaris* (Sw.) D. Don ex DC. | Melastomataceae |  | n/a | | HM446827 | HM446959 |
| *Miconia laevigata* (L.) D. Don | Melastomataceae |  | n/a | | HM446828 | HM446960 |
| *Miconia mirabilis* (Aubl.) L.O. Williams | Melastomataceae |  | n/a | | HM446829 | HM446961 |
| *Miconia prasina* (Sw.) DC. | Melastomataceae |  | n/a | | HM446830 | HM446962 |
| *Miconia racemosa* (Aubl.) DC. | Melastomataceae |  | n/a | | HM446831 | HM446963 |
| *Miconia tetrandra* (Sw.) D. Don ex G. Don | Melastomataceae |  | n/a | | HM446832 | HM446964 |
| *Micropholis garciniifolia* Pierre | Sapotaceae |  | HM446713 | | HM446833 | HM446965 |
| *Micropholis guayanensis* (A. DC.) Pierre | Sapotaceae |  | HM446714 | | HM446834 | HM446966 |
| *Myrcia deflexa* (Poir.) DC. | Myrtaceae |  | HM446715 | | HM446835 | HM446967 |
| *Myrcia fallax* DC. | Myrtaceae |  | HM446716 | | HM446836 | HM446968 |
| *Myrcia leptoclada* DC. | Myrtaceae |  | HM446717 | | HM446837 | HM446969 |
| *Myrcia splendens* (Sw.) DC. | Myrtaceae |  | HM446718 | | HM446838 | HM446970 |
| *Myrsine coriacea* (Sw.) R. Br. ex Roem. & Schult. | Primulaceae |  | n/a | | HM446839 | n/a |
| *Nectandra antillana Meisn.* | Lauraceae |  | n/a | | GQ981812* | HM446971 |
| *Ochroma pyramidale* (Cav. ex Lam.) Urb. | Malvaceae |  | HM446719 | | HM446840 | n/a |
| *Ocotea floribunda* (Sw.) Mez | Lauraceae |  | HM446720 | | HM446841 | HM446972 |
| *Ocotea leucoxylon* (Sw.) Laness. | Lauraceae |  | HM446721 | | HM446842 | HM446973 |
| *Ocotea moschata* (Meisn.) Mez | Lauraceae |  | HM446722 | | HM446843 | HM446974 |
| *Ocotea sintensis* (Mez) Alain | Lauraceae |  | HM446723 | | HM446844 | HM446975 |
| *Ocotea spathulata* Mez | Lauraceae |  | HM446724 | | HM446845 | HM446976 |
| *Ormosia krugii* Urb. | Fabaceae |  | HM446725 | | HM446846 | HM446977 |
| *Oxandra laurifolia* (Sw.) A. Rich. | Annonaceae |  | HM446726 | | HM446847 | HM446978 |
| *Palicourea riparia* Benth. | Rubiaceae |  | HM446727 | | HM446848 | HM446979 |
| *Piper aduncum* L. | Piperaceae |  | HM446728 | | HM446849 | HM446980 |
| *Piper blattarum* Spreng. | Piperaceae |  | HM446729 | | HM446850 | HM446981 |
| *Piper glabrescens* (Miq.) C. DC. | Piperaceae |  | HM446730 | | HM446851 | HM446982 |
| *Piper hispidum* Sw. | Piperaceae |  | n/a | | HM446852 | HM446983 |
| *Piper umbellatum* L. | Piperaceae |  | n/a | | HM446853 | HM446984 |
| *Pisonia subcordata* Sw. | Nyctaginaceae |  | HM446731 | | HM446854 | HM446985 |
| *Pleodendron macranthum* Teigh. | Canellaceae |  | HM446732 | | HM446855 | HM446986 |
| *Prestoea acuminata* (Willd.) H.E. Moore | Arecaceae |  | HM446733 | | HM446857 | HM446987 |
| *Pseudolmedia spuria* (Sw.) Griseb. | Moraceae |  | HM446734 | | HM446858 | HM446988 |
| *Psychotria berteroana* DC. | Rubiaceae |  | HM446735 | | HM446859 | HM446989 |
| *Psychotria brachiata* Sw. | Rubiaceae |  | HM446736 | | HM446860 | HM446990 |
| *Psychotria deflexa* DC. | Rubiaceae |  | HM446737 | | HM446861 | HM446991 |
| *Psychotria grandis* Sw. | Rubiaceae |  | HM446738 | | HM446862 | HM446992 |
| *Pterocarpus officinalis* Jacq. | Fabaceae |  | HM446739 | | HM446863 | HM446993 |
| *Pterocarpus rohrii* Vahl | Fabaceae |  | n/a | | GQ981862* | n/a |
| *Quararibea turbinate* (Sw.) | Malvaceae |  | HM446740 | | HM446864 | HM446994 |
| *Rauvolfia nitida* Lam. | Apocynaceae |  | n/a | | DQ660663* | n/a |
| *Rondeletia portoricensis* J.C. Krug and Urb. | Rubiaceae |  | HM446741 | | HM446866 | HM446996 |
| *Roystonea borinquena* O.F. Cook | Arecaceae |  | HM446742 | | HM446867 | HM446997 |
| *Samyda spinulosa* Vent. | Salicaceae |  | HM446743 | | HM446868 | HM446998 |
| *Sapium laurocerasus* Desf. | Euphorbiaceae |  | n/a | | HM446869 | HM446999 |
| *Schefflera morototoni* (Aubl.) Maguire, Steyerm. & Frodin | Araliaceae |  | HM446744 | | HM446870 | HM447000 |
| *Simarouba amara* Aubl. | Simaroubaceae |  | n/a | | HM446871 | HM447001 |
| *Sloanea berteroana* Choisy ex DC. | Elaeocarpaceae |  | HM446745 | | HM446872 | HM447002 |
| *Spathodea campanulata* P. Beauv. | Bignoniaceae |  | HM446746 | | HM446873 | HM447003 |
| *Swietenia macrophylla* King | Meliaceae |  | HM446747 | | HM446874 | HM447004 |
| *Symplocos martinicensis* Jacq. | Symplocaceae |  | n/a | | HM446875 | HM447005 |
| *Syzygium jambos* (L.) Alston | Myrtaceae |  | HM446748 | | HM446876 | HM447006 |
| *Tabebuia heterophylla* (DC.) Britton | Bignoniaceae |  | n/a | | HM446877 | HM447007 |
| *Ternstroemia luquillensis* Krug & Urb. | Theaceae |  | n/a | | AF380065.1* | n/a |
| *Tetragastris balsamifera* (Swartz) Oken | Burseraceae |  | HM446749 | | HM446878 | HM447008 |
| *Trema micrantha* L. |  |  | n/a | | U03844.1* | n/a |
| *Trichilia pallida* Sw. | Meliaceae |  | HM446750 | | HM446879 | HM447009 |
| *Turpinia occidentalis* (Sw.) G. Don | Staphyleaceae |  | HM446751 | | HM446880 | HM447010 |
| *Urera baccifera* (L.) Gaudich. ex Wedd. | Urticaceae |  | HM446752 | | HM446881 | HM447011 |
| *Vitex divaricata* Sw. | Lamiaceae |  | n/a | | U78716* | HM447012 |
| *Xylosma schwaneckeana* Urb. | Salicaceae |  | HM446753 | | HM446882 | HM447013 |
| *Zanthoxylum martinicense* (Lam.) DC. | Rutaceae |  | HM446754 | | HM446883 | HM447014 |
|  |  |  |  | |  |  |
