# Supplementary material for: Advances in the Use of DNA Barcodes to Build a Community Phylogeny for Tropical Trees in a Puerto Rican Forest Dynamics Plot
Source: PLoS One. 2010 Nov 9;5(11):e15409. doi: 10.1371/journal.pone.0015409 (PMC2976767; doi:10.1371/journal.pone.0015409)
Supplement: Table S2 — Primer pairs for barcode regions rbcLa, matK, and trnH-psbA. (DOC) [file pone.0015409.s002.doc]

| **Marker** | **Primer** | **Sequence (5’ 🡪 3’)** | **Tm** | **Reference** |
| --- | --- | --- | --- | --- |
| ***rbcLa*** | SI_For | ATGTCACCACAAACAGAGACTAAAGC | 55C | 1 |
|  | SI_Rev | GTAAAATCAAGTCCACCRCG |  |  |
| ***matK*** | KIM 3F | CGTACAGTACTTTTGTGTTTACGAG | 49C | 2 |
|  | KIM 1R | ACCCAGTCCATCTGGAAATCTTGGTTC |  |  |
|  | 1329 | TCTAGCACACGAAAGTCGAAGT | 49C | 3 |
|  | 320 | CGATCTATTCATTCAATATTTC |  |  |
|  | Kew 5R | GTTCTAGCACAAGAAAGTCG | 48C | 4 |
|  | Kew XF | TAATTTACGATCAATTCATTC |  | 5 |
| ***trnH-psbA*** | *psbA*3’f | GTTATGCATGAACGTAATGCTC | 55C | 6 |
|  | *trnH* | CGCGCATGGTGGATTCACAATCC |  | 7 |

References:

1. Kress WJ, Erickson DL, Jones FA, Swenson NG, Perez R, et al. (2009) Plant DNA Barcodes and a Community Phylogeny of a Tropical Forest Dynamics Plot in Panama. *PNAS*, 106, 18621-18626.

2. CBOL Plant Working Group (2009) A DNA barcode for land plants. *PNAS* 106, 12794-12797

3. Cuénoud P, Savolainen V, Chatrou LW, Powell M, Grayer RJ, *et al*. (2002) Molecular phylogenetics of Caryophyllales based on nuclear 18S rDNA and plastid rbcL, atpB, and matK DNA sequences. *Am J Bot* 89, 132–144.

4. <http://www.kew.org/barcoding/protocols.html>.

5. Soltis DE, Tago-Nakazawa M, Xiang Q-Y, Kawano S, Murata J, *et al.* (2001) Phylogenetic relationships and evolution in *Chrysosplenium* (Saxifragaceae) based on matK sequence data. *Am J Bot* 88, 883-893.

6. Sang T, Crawford DJ, Stuessy TF (1997) Chloroplast DNA phylogeny, reticulate evolution, and biogeography of *Paeonia* (Paeoniaceae). *Am J. Bot* 84, 1120–1136.

7. Tate JA, Simpson, BB (2003) Paraphyly of *Tarasa* (Malvaceae) and diverse origins of the polyploid species. *Syst Bot* 28, 723–737.
